# Targeting the MHC Ligandome by Use of TCR-Like Antibodies

**DOI:** 10.3390/antib8020032

**Published:** 2019-05-09

**Authors:** Lene Støkken Høydahl, Rahel Frick, Inger Sandlie, Geir Åge Løset

**Affiliations:** 1 Department of Immunology, University of Oslo and Oslo University Hospital, N-0372 Oslo, Norway; l.s.hoydahl@medisin.uio.no (L.S.H.); rahel.frick@medisin.uio.no (R.F.); inger.sandlie@ibv.uio.no (I.S.); 2 KG Jebsen Coeliac Disease Research Centre, University of Oslo, N-0372 Oslo, Norway; 3 Department of Biosciences, University of Oslo, N-0316 Oslo, Norway; 4 Nextera AS, N-0349 Oslo, Norway

**Keywords:** TCR-like antibodies, epitope, MHC, antigen-specific therapy

## Abstract

Monoclonal antibodies (mAbs) are valuable as research reagents, in diagnosis and in therapy. Their high specificity, the ease in production, favorable biophysical properties and the opportunity to engineer different properties make mAbs a versatile class of biologics. mAbs targeting peptide–major histocompatibility molecule (pMHC) complexes are often referred to as “TCR-like” mAbs, as pMHC complexes are generally recognized by T-cell receptors (TCRs). Presentation of self- and non-self-derived peptide fragments on MHC molecules and subsequent activation of T cells dictate immune responses in health and disease. This includes responses to infectious agents or cancer but also aberrant responses against harmless self-peptides in autoimmune diseases. The ability of TCR-like mAbs to target specific peptides presented on MHC allows for their use to study peptide presentation or for diagnosis and therapy. This extends the scope of conventional mAbs, which are generally limited to cell-surface or soluble antigens. Herein, we review the strategies used to generate TCR-like mAbs and provide a structural comparison with the analogous TCR in pMHC binding. We further discuss their applications as research tools and therapeutic reagents in preclinical models as well as challenges and limitations associated with their use.

## 1. Introduction

Antibodies and T-cell receptors (TCRs) are highly diverse antigen-specific receptors expressed by B cells and T cells, respectively. Antibodies are usually directed against cell-surface or soluble target antigens. In contrast, TCRs recognize target antigens in the form of peptide presented on major histocompatibility (MHC) class I (MHCI) and MHC class II (MHCII) molecules. Thus, antibodies and TCRs are equipped to sample and specifically bind to almost any structure representing a threat to the organism, either outside or inside the cell. A key feature that distinguishes the binding interaction of antibodies and TCRs is the difference in affinity for their ligands; while TCRs generally have affinities in the range of 1–100 µM after thymic selection [1,2], antibodies exhibit affinities in the nanomolar and sub-nanomolar range [3,4].

The use of soluble TCRs as research reagents and as therapeutics is hampered by low target affinity and challenges related to expression [5]. Still, strategies to increase TCR avidity and/or affinity have enabled studies of endogenous peptide presentation, assessment of peptide–MHC (pMHC) half-life, and estimates of the number of pMHC complexes on the cell surface [6,7,8,9,10,11]. In recent years, TCR-engineered T cells have emerged as promising therapeutics, particularly in cancer treatment [12,13]. Here, the use of T cells harboring either native TCRs or TCRs engineered for high affinity are explored [13,14]. However, increased target affinity, at least at cellular level, appears to come at the cost of unpredictable cross-reactivity [15,16,17], which has led to severe toxicity in vivo [18,19]. Engineered high-affinity, soluble TCRs, on the other hand, have shown great promise in preclinical models both regarding specificity and potency, but are still in early clinical development as a novel class of therapeutics [20,21,22,23].

The challenges associated with soluble TCR expression and low target affinity have motivated development of antibodies with TCR-like specificity. The versatility and specificity of antibodies have made them the most valuable research reagents of biology, and their utility extends further to diagnosis and therapy [24]. Antibodies binding pMHC, often referred to as TCR-like antibodies (or TCR mimic (TCRm) antibodies by some groups), combine the ability to target specific pMHC complexes with the favorable properties of antibodies. Whereas monoclonal antibodies (mAb) used in therapy usually bind cell surface or soluble antigens, TCR-like mAbs provide a complementary strategy by targeting intracellular or extracellular antigens presented on MHC. In this review, we focus on the generation of such TCR-like mAbs, how they bind pMHC compared to TCRs and their use as research tools and in therapy.

## 2. Peptide Presentation on MHC

The MHC ligandome represents all the peptides presented on MHC molecules. This peptide repertoire can be viewed as a snapshot of all proteins expressed by and endocytosed by the presenting cells. In brief, there are two major groups of peptides presented on MHC, namely those found on MHCI and MHCII, respectively. Those peptides presented on MHCI are normally proteolytic fragments of endogenously produced proteins from the cell displaying the pMHC, whereas the peptides found on MHCII usually originate from extracellular material taken up and processed by the pMHC-displaying cell through a variety of mechanisms [25,26]. In recent years, it has become increasingly clear that there may be an extensive cross-talk between these two pMHC compartments [27].

The MHC molecules are highly polymorphic with the majority of variation clustering in the region forming the peptide-binding groove that exhibits distinct requirements for shape and biophysical properties of the interacting peptides [2]. Conserved architectural features that distinguish MHCI and MHCII separate their respective peptide ligandomes into distinct peptide lengths. The pMHCI compartment has a rather strict preference for short nonameric peptides, in contrast to the pMHCII ligandome, which may comprise peptides with lengths even above 40 amino acids [28]. The peptides bind MHC by anchoring amino acid side chains into pockets in this groove, whereas other residues are exposed for TCR interactions. Although each MHC molecule can accommodate a vast number of peptides, different alleles prefer certain peptide sequences, and there is great interest in deciphering these preferences to better understand health and disease [29,30]. As a result, the composition of the peptide-binding groove shapes the peptide repertoire presented by a given MHC, which forms the molecular basis for MHC association seen in diseases ranging from autoimmunity to cancer and infection [31].

## 3. Antibodies with Specificity for pMHC Molecules

Naturally occurring antibodies with TCR-like specificity are thought to be extremely rare. Thus, the generation of TCR-like mAbs relies on engineering techniques first made possible by the introduction of the hybridoma technology [32]. In subsequent years, display technologies have been increasingly used as an alternative to immunization, allowing for tailored selection of binders. An additional key aspect accounting for the increase in successful isolation of specific binders has been development of methods in recombinant MHC technology. This has been fundamental in enabling tailored selection strategies, including screening on different peptide variants, allowing one to single out the rare clones with true peptide-specificity and MHC restriction.

In order for TCR-like mAbs to be broadly applicable, e.g., in therapy, there has been a focus on targeting disease-relevant peptides presented on widely expressed MHC variants. In the human population, human leukocyte antigen (HLA)-A2 and HLA-DP4 stand out as the most prevalent MHCI and MHCII molecules, respectively [33,34]. The importance of CD8-restricted responses in cancer immune control and the relative ease in manufacturing recombinant pMHCI molecules likely accounts for the bias towards TCR-like Abs against HLA-A2 peptide complexes (Table 1). In the MHCII compartment, allele frequencies in the general population are of less importance, as the HLA predisposition towards autoimmune disease largely dictates which complexes are the targets of attention (Table 2) [31]. Table 1 and Table 2 list all TCR-like mAbs reported to date to the best of our knowledge, split into MHCI and MHCII specificities, respectively.

### 3.1. TCR-Like mAbs via Hybridoma Technology

Hybridoma technology has been used to generate antibodies against both MHCI and MHCII, and the first TCR-like mAbs were produced using this method [35,36,79,84]. Antibody-secreting hybridomas are generated by immunization of mice with soluble, recombinant antigen, or cells expressing the desired antigen, before isolation of B cells and fusion with myeloma cells. Subsequent identification of antigen-specific clones usually requires screening of hundreds or even thousands of clones [39,81,84]. Additionally, such factors as low immunogenicity, few unique clones due to immunodominance, and poor control of fine-specificity have been hampering antibody discovery through this route [57,71]. An advantage of hybridoma technology is the potential for natural affinity maturation, which often results in higher affinity mAbs.

The efficiency and success of the hybridoma method has been greatly improved by use of purified, recombinant protein for immunization and in vitro screening using multiple protein variants to test specificity. This is also true for MHC-restricted TCR-like mAbs, but their isolation is still challenging. However, the success rate has improved by combining immunization using high-quality pMHC protein with enrichment of antigen-specific B cells before generation of hybridomas [69,78]. Another approach to increase the frequency of specific B cells is the use of mice transgenic for a TCR β-chain derived from a TCR with specificity for the target antigen for mAb discovery (I-A^d^ presenting an epitope from the Leishmania homologue of activated C kinase (LACK) antigen of the parasite *Leishmania major*) [80]. The rationale behind this was that CD4 T cells from such mice could efficiently provide help and rescue antigen-specific B cells. Only one antigen-specific clone was identified, however.

### 3.2. TCR-Like mAbs via Phage Display

In antibody phage display, sequences encoding either Fab or scFv are genetically linked to a phage coat protein resulting in display of the fusion protein on the surface of the phage, ensuring a physical genotype–phenotype coupling. A display library may contain more than 10^10^ unique binders [92]. There are three main types of antibody libraries: immune, naïve and synthetic [93]. These libraries can be selected on antigen in a process termed biopanning to retrieve specific binders. By performing multiple rounds of selection, modulating both positive and negative selection steps as well as the stringency (e.g., washing, antigen competition, elution), it is possible to direct the clonal output in the desired direction regarding the fine-specificity and other properties of the selected mAbs [94,95,96].

Phage display has contributed greatly to the field of monoclonal antibody generation and has reduced the cost and time needed compared to hybridoma technology [97,98]. In addition, antibody phage display libraries offer a rich source of fully human antibodies alleviating cross-species issues important for, e.g., therapeutic use [99]. A major step forward with regard to TCR-like mAb generation was made when Andersen et al. combined immunization and display technology [38]. By immunizing mice with target pMHC followed by generation of Fab phage libraries, antibodies against both MHCI and MHCII were successfully isolated [38,87].

In 2000, the first TCR-like mAb isolated directly from a naïve human phage library was reported, which recognized HLA-A1 with a peptide from the tumor-derived antigen MAGE-A1 [40]. Despite utilizing a large library (3.7 × 10^10^ independent clones), the candidate antibody was of low affinity, and subsequent affinity maturation allowed for more efficient pMHC detection and target cell killing in vitro [40,42,100]. Since then, many TCR-like mAbs have been isolated (Table 1 and Table 2).

Almost all TCR-like mAbs generated by phage display have been isolated from either immune libraries or from libraries constructed from endogenous antibody repertories. An exception is the single domain antibody (sDAb) A2/Ab (or clone3), against the heat shock protein 16 kDa (HSP16) antigen of *Mycobacterium tuberculosis* presented on HLA-A2 [73]. Although the specificity of this clone remains to be demonstrated, it is noteworthy that it was isolated from an sDAb built on human *IGHV3-23* (DP-47) with diversity introduced in the complementarity-determining regions (CDR) 1–3 only. This indicates that both HLA restriction and peptide specificity can be conferred by a single Ig domain [73]. Other exceptions are the mAbs against HLA-A2/KRAS, HLA-A2/EGFR, HLA-A1/Her2, HLA-A2/Calreticulin and HLA-A2/WT1 (clone45) that are derived from VH/VL single-framework libraries with variations introduced in the CDRs [61,68,71]. Such single-framework libraries represent a compromise between the broad sequence space offered by libraries built on endogenous antibody repertoires and the more favorable manufacturability offered by libraries built on certain frameworks characterized by superior biophysical properties [92]. Given the anticipated advantages of such libraries, it is rather striking that most pMHC binders are still isolated from libraries built on endogenous variable gene repertoires.

### 3.3. TCR-Like mAbs from Other Display Platforms and Methodologies

The yeast display platform was described more than 20 years ago and is now a well-established display platform along with phage display [101]. A major advantage with yeast display is that the use of a eukaryotic expression host enables complex glycosylation and folding quality control mechanisms. Even more importantly, yeast display merges the combinatorial diversity with flow cytometric sorting of desired specificities, which allows for rare events to efficiently be singled out. However, such sorting is critically dependent on the availability of a fluorescently labeled high-quality target. Additionally, library size is usually orders of magnitude lower than what is possible with phage display.

As for phage display, antibody libraries of naïve, immune or synthetic origin can be displayed on yeast. Still, to our knowledge, there is only one TCR-like mAb generated by yeast display [68]. Here, a scFv (originally discovered through phage selections) specific for a peptide derived from the cancer antigen Wilms tumor protein 1 (WT1) presented on HLA-A2 was affinity-matured using random mutagenesis in combination with yeast display selection on pMHC multimers, resulting in a 100-fold affinity improvement yet retained specificity [68].

## 4. pMHC–mAb Structures

**Figure 1:**
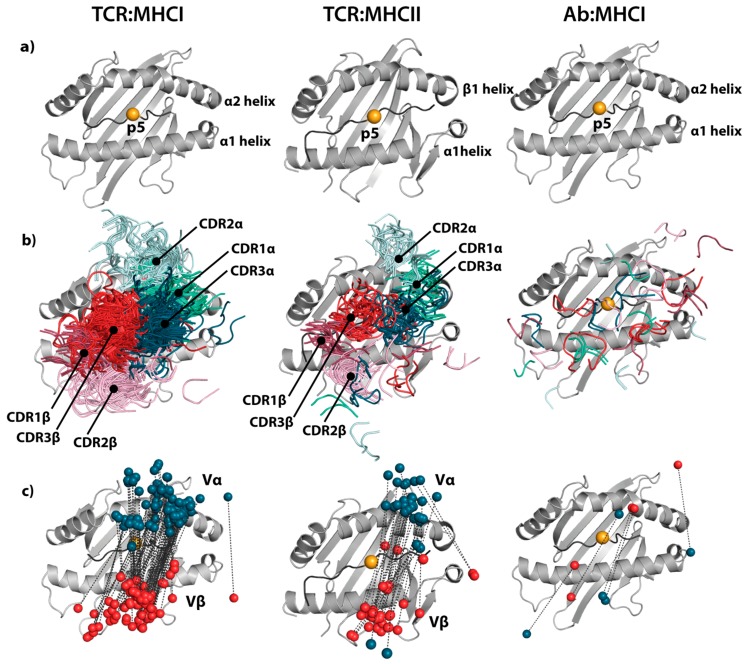
Overlay of all publicly available co-crystal structures of major histocompatibility complex (MHC) molecules with T-cell receptors (TCRs) or TCR-like mAbs. To illustrate the conservation in binding mode between TCRs and peptide-MHC (pMHC), we collected all co-crystal structures of human α/β TCRs in complex with MHC using the STCRDab [107]. We obtained 103 and 33 complexes with MHC class I (MHCI) and MHC class II (MHCII), respectively. In addition, we collected the five available co-crystal structures of TCR-like mAbs in complex with MHCI from the Protein Data Bank (PDB). Representative pMHC complexes (PDB IDs: 1AO7, 4OZF, 1W72) are illustrated and the central p5 position of the peptides is highlighted (**a**). The complementarity-determining region (CDR) loops of TCRs or antibodies are represented as cartoons and annotated (**b**). The centers of mass of the variable domains are represented as spheres (red: variable β/variable heavy, dark teal: variable α/variable light) and connected with dashed lines to illustrated orientations (**c**).

TCRs are the natural binding partners for pMHC complexes. Interestingly, the orientation of the TCR relative to the pMHC complex has been found to be strikingly conserved [102], as illustrated in Figure 1. Generally, TCRs bind in a conserved diagonal mode, positioning Vα over the *N*-terminal half of the peptide and Vβ over the *C*-terminal half [2]. The germline-encoded CDR1 and CDR2 loops are positioned over the α-helices that form the rim of the peptide-binding groove in the MHC molecule. The CDR3 loops are focused on the peptide, typically centered on the p5 position, and thus provide the major contribution towards peptide specificity. Even relatively small deviations from this canonical binding mode have been associated with non-canonical peptides [103,104] and inability to induce TCR signaling [105], but exceptions deviating strongly from the canonical binding orientation exist [106].

For MHCI, the only clear exception from this general docking mode is the SB47 TCR contacting HLA-B*35-08 with a bulged 13-residue-long viral peptide (PDB: 4JRY, [108]). This TCR is shifted towards the peptide *N*-terminus compared to the canonical orientation. For MHCII, again almost all TCRs dock in the conserved orientation across the peptide-binding groove. Two TCRs (FS17 and FS18, PDB: 4Y1A and 4Y19) from human induced T regulatory cells in complex with HLA-DR4 presenting an insulin peptide are exceptions [106]. These two TCRs adopt a conformation that is rotated by 180° and shifted towards the MHC α-chain. Another two structures are markedly tilted away from the canonical orientation towards the peptide *N*-terminus and the MHC β-chain (PDB: 1YMM [109] and 2WBJ [110]). These involve the same Ob.1A12 TCR bound to an MBP self-peptide and an *Escherichia coli*-derived peptide on HLA-DR2b, respectively.

It is striking that almost all TCRs with solved co-crystal structures have highly similar binding modes, and the reasons for this are still debated [111,112]. One explanation is that the conserved orientation is solely the result of thymic selection [113,114], where only those T cells that have a TCR:MHC interaction that allows for CD4 or CD8 co-receptor engagement are recruited to the naïve repertoire. Orientations deviating from the canonical binding mode may fail to induce TCR signaling due to lack of MHC specificity or lack of co-receptor engagement [1]. Another explanation is that TCRs are predisposed to interact with certain MHC alleles via germline-encoded features in their CDR1 and CDR2 loops [115,116,117]. These residues then form interactions with the MHC helices, which in turn have acquired complementary residues via co-evolution. This hypothesis is supported by a recent large-scale expression quantitative trait loci (eQTL) analysis [117], where trans-associations between TCR V genes and the MHC locus were observed. The germline-encoded residues are then presumed to bias the TCR repertoire towards a diagonal orientation even before thymic selection.

Antibodies are primarily directed toward non-self-components, such as exogenous pathogenic structures, and are purely selected based on their ability to bind target specifically, and are independent of restrictions imposed by, e.g., co-receptors, such as CD4 and CD8, that are necessary for signaling. For these reasons, antibodies can take on diverse binding conformations to achieve specificity, which is also seen by the different docking modes utilized by TCR-like mAbs to bind pMHC (Figure 1) [118]. Crystallization and mutagenesis studies of TCR-like mAbs show that they recognize pMHC in a way similar to the corresponding TCRs, or utilize non-canonical binding modes [41,50,52,87,119,120,121]. That there is no particular inherent interaction mode between antibodies and MHC molecules is reflected in a lack of any apparent eQTL association [117]. Comparison of the five available co-crystal structures of TCR-like mAbs bound to pMHC (PDB: 1W72 [41], 3CVH [119], 3GJF, 3HAE [52], 4WUU [66]), confirms that antibodies exhibit more diverse binding modes. Two of the antibodies (PDB: 3GJF, 3HAE) show highly similar orientations to each other; however, they differ at only the 3 and 6 amino acid positions in the light and heavy chain, respectively, and they are specific for the same epitope. Their binding mode is similar to the canonical orientation of TCRs, where the antibody VL domain corresponds to the TCR Vβ domain and the antibody VH domain corresponds to the TCR Vα domain. All other structures differ significantly from each other and from the canonical binding mode of TCRs. However, all antibodies rely on their CDR3 loops for direct interactions with the peptide, albeit to different degrees.

## 5. TCR-Like mAbs as Tools to Study Specific Peptide-Presentation

Knowledge about the presentation of antigenic peptides that drive a specific immune response is fundamental for elucidating disease mechanisms. Important aspects include the levels of peptide-presentation as well as the phenotypic characteristics of the peptide-presenting cells at different stages of a disease. TCR-like mAbs have been used as reagents to detect and quantify peptide-presentation in vitro, offering an alternative approach to labeled peptides, mass spectrometry or T-cell activation. For example, using a TCR-like mAb with specificity for I-E^k^ presenting a moth cytochrome c-derived peptide (MCC), it was determined that 200–400 pMHCII complexes per model antigen presenting cell (APC) were necessary and sufficient to induce a minimal T-cell response [85]. Importantly, similar estimates were previously obtained in the I-E^k^/hen egg lysozyme (HEL) model system using labeled peptides and hybridoma T cells [122]. For quantification of pMHCI, a TCR-like mAb against HLA-A2/MAGE3 indicated that as few as 10 complexes per model APC are required for the cytotoxic activity of primary human T-cell clones [57]. This is comparable to previous estimates of 3–10 pMHCI complexes being sufficient to induce cytotoxicity in two murine H-2K^b^-restricted model systems [123].

Quantification of pMHC levels using TCR-like mAbs has not only illuminated the minimum number of pMHCs required to trigger T-cells activation, and thus confirmed estimates inferred from T-cell activation data, but also led to novel insights into peptide presentation. Sim et al. generated a panel of TCR-like mAbs to map the expression hierarchy of the Epstein-Barr virus (EBV) epitopes derived from LMP1, LMP2A and EBNA1 presented on HLA-A2 on both cell lines and EBV-associated tumor biopsies. A surprising discordance between pMHC density and frequencies of associated cytotoxic T lymphocyte (CTL) responses was seen [69]. Further, epitope density was also shown to affect the therapeutic efficacy of the TCR-like mAbs, as only the mAb targeting EBNA1 could delay weight loss and improve survival of NSG (NOD SCID Il2rg^-/-^) mice injected with EBV-infected B lymphoblastoid cell lines [124]. Notably, the EBNA1-specific mAb did not have higher affinity than the other mAbs, but the higher EBNA1 pMHC density was suggested to explain the superior efficacy [69].

Peptide-presentation has also been studied in the context of infection. Weidanz et al. isolated a mAb, 4F7, targeting a self-peptide derived from eIF4G, suspected to be differentially presented in healthy and HIV-1 infected cells, in complex with HLA-A2 [74]. 4F7 was used to directly study peptide-presentation on cells, which revealed that the self-peptide on HLA-A2 was indeed upregulated 3-fold in infected cells. Muraille et al. generated an mAb specific for an antigen derived from the parasite *Leishmania major* bound to an MHCII molecule [80]. Flow cytometry and electron microscopy experiments revealed that, while intracellular pMHC complexes are found in different groups of APCs, cell-surface expression was exclusively seen on dendritic cells (DCs).

TCR-like mAbs targeting MHCII have mostly been generated towards complexes associated with autoimmunity (Table 2). The MHCII locus is often the primary predisposing genetic factor; however, in most autoimmune diseases, the relevant autoantigen(s) and the epitopes recognized by pathogenic T cells are unknown or poorly characterized [31]. The TCR-like mAb MK16 is specific for a myelin basic protein (MBP)-derived peptide, one of the proposed autoantigens in multiple sclerosis (MS), bound to the disease-associated HLA-DR2b molecule and was used to assess peptide-presentation in patient tissue [87]. Immunohistochemistry on tissue sections from MS lesions confirmed presentation of the MBP-derived peptide and identified microglia/macrophages as the dominant APC. Later on, MBP presentation by microglia and DCs was also suggested to play a role in experimental autoimmune encephalomyelitis (EAE) in susceptible HLA-DR2b humanized mice [125]. Here, these cells co-localized with CD4 T cells, implicating a direct role in activation of MBP-reactive T cells. Similarly, the TCR-like mAb A12 was used to assess if the proposed autoantigen, human cartilage glycoprotein-39 (HC gp-39), could be found presented on HLA-DR4.1 in inflamed synovial lesions of type 1 rheumatoid arthritis (RA) patients [90]. Indeed, DCs present in the synovial tissue from RA patients presented the HC gp-39-derived peptide, demonstrating that this potential autoantigen is presented at the site of inflammation [90,126]. Moreover, positive staining correlated with more extensive synovial inflammation in a cohort of 65 patients [126].

We have recently developed a TCR-like mAb specific for one of the immunodominant epitopes of wheat gluten presented on HLA-DQ2.5, a pMHC complex characteristic of the autoimmune condition celiac disease (CeD) [91]. Flow cytometric analysis of single-cell suspensions generated from gut biopsies, the site of the tissue destruction, surprisingly showed that plasma cells were the most abundant cell type presenting the gluten peptide in patients. Thus, the use of this mAb has demonstrated a potential new role of plasma cells beyond antibody secretion, implying APC capability.

## 6. TCR-Like mAbs as Therapeutics

**Figure 2:**
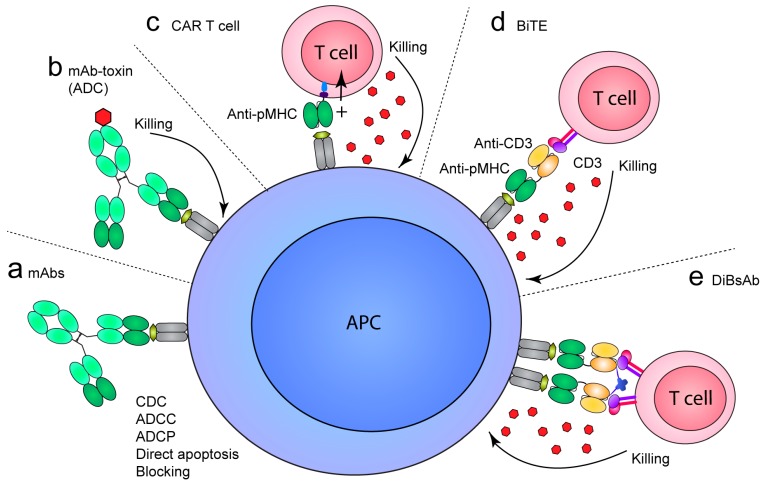
Potential modes of action of TCR-like mAbs in cancer, infection and autoimmunity. (**a**) TCR-like mAbs used in a classical antibody format, usually a full-length IgG, where binding to pMHC blocks T-cell accessibility (autoimmunity) or induces direct apoptosis. Alternatively, Fc-mediated effector functions, such as complement-dependent cytoxicity (CDC), antibody-dependent cellular cytotoxicity (ADCC) or antibody-dependent cellular phagocytosis (ADCP), can lead to targeted destruction of the peptide-presenting cell. (**b**) Targeted delivery of toxic payloads by antibody–drug conjugates (ADCs), where effector molecules, such as cytokines, toxins or radioactive substances, are coupled to an antibody format. Internalization of the complex leads to cell death. (**c–e**) Various strategies exist to redirect cytotoxic cells to a target cell. Binding triggers release of perforin and granzymes inducing apoptosis of the target cell in a co-receptor-independent manner. (**c**) Chimeric antigen receptor (CAR) T cells are redirected to peptide-presenting cells via the scFv fragment derived from a TCR-like mAb. (**d**,**e**) Bispecific protein formats, such as (**d**) bispecific T-cell engagers (BiTEs) and (**e**) bivalent formats illustrated by the dimeric bispecific T-cell-engaging tandem scFv antibodies (DiBsAbs), indirectly recruit T cells by bridging CD3 on T cells and natural killer (NK) T cells and the peptide-presenting cell.

Besides the value as research reagents, TCR-like mAbs are promising therapeutics since they specifically target cells presenting pathogenic peptides. As such, they may be used to selectively kill transformed cells in cancer or viral infection (MHCI), but also to target APCs presenting self-peptides in autoimmunity to prevent pathogenic T-cell responses (MHCII). The ability to selectively target cell populations presenting a particular peptide reduces the risk of side effects associated with broadly acting drugs. For example, treatment of hematologic malignancies and autoimmune disease through targeting of lineage markers, such as CD20 and CD52, results in removal of lymphocyte populations rendering the patient immunocompromised and, e.g., susceptible to opportunistic infections. In common for all formats of the TCR-like mAb specificity, only those B cells presenting the epitope of interest will be targeted, sparing the other cells that are important for maintaining immune protection. Whereas mAbs are widely used to treat a range of diseases, TCR-like mAbs have not yet been approved for therapeutic use. However, various strategies are explored, and these can be broadly grouped into two categories: 1) strategies utilizing classical, soluble antibody formats to, e.g., block pMHC accessibility, deliver a toxic payload or induce Fc-mediated recruitment of effector cells or molecules depending on the antibody isotype (Figure 2a,b); and 2) strategies utilizing formats to redirect cytotoxic cells or bridge the cytotoxic cell with the peptide-presenting cell (Figure 2c–e). These cytotoxic cells are usually CD3 cells, which are predominantly T cells and natural killer (NK) T cells. These can be engineered to express chimeric antigen receptors (CARs), which combine signaling domains of TCRs and Fv regions of antibodies to confer target specificity. Alternatively, cytotoxic T cells can be recruited indirectly via bispecific molecules, such as bispecific T-cell engagers (BiTEs) or related formats specific for the target cells and CD3 on the T cells and NK T cells.

### 6.1. Cancer

The proof-of-principle of targeting cytotoxic drugs to cancer cells by use of TCR-like specificities was provided in 1997 (Figure 2b). Here, the genetic fusion of the murine MHCI-restricted Fab13.4.1, specific for K^k^ with a hemagglutinin (HA) peptide, to Pseudomonas exotoxin (PE38) was shown to induce specific killing of influenza virus-infected cells in vitro [127]. Later, Klechecsky et al. demonstrated the in vivo potential of TCR-like specificities as antibody–drug conjugates (ADCs) [54]. Here, Fab fragments specific for HLA-A2 in complex with peptides derived from MART-1 (Fab CLA12) or gp100 (Fab 2F1) fused to a modified Pseudomonas exotoxin (PE38KDEL) were shown to trigger rapid internalization of pMHC upon binding to peptide-loaded cells and to exert cytotoxic activity towards melanoma cells presenting endogenous peptides. Importantly, when injected into NOD SCID β2M-deficient mice with established melanoma, both ADCs demonstrated anti-tumor activity as seen by a reduction in tumor growth compared to a control conjugate.

Several TCR-like mAbs target the HLA-A2-restricted epitope, “RMF”, derived from the intracellular transcription factor WT1, which is overexpressed in a range of leukemias and solid cancers [65,68]. As a hIgG1 molecule, the TCR-like mAb ESK1 demonstrated potent killing in vitro in antibody-dependent cellular cytotoxicity (ADCC) assays (but not complement-dependent cytoxicity (CDC) or antibody-dependent cellular phagocytosis (ADCP)) [65]. Such Fc-mediated effector functions were also demonstrated in an acute lymphoblastic leukemia (ALL) xenograft model in NSG mice. To rule out T cell or NK T cell-mediated killing, peripheral blood mononuclear cells (PBMCs) were depleted of CD3 and CD34 cells before injection of effector cells along with ESK1 hIgG1. Indeed, prolonged survival was seen and the therapeutic effects were shown to be Fc-dependent, as absence of the Fc region did not prolong survival. In line with this, ESKM, a hIgG1 variant containing Fc glycol modifications resulting in improved binding to activating FcγRs, was more potent at ADCC in vitro and also demonstrated improved survival in the leukemia mouse model compared to the unmodified mAb [65,128].

The success of strategies redirecting cytotoxic cells to kill target cells, such as CAR T cells or soluble protein formats, such as the bispecific T-cell engagers (BiTEs), for treatment of hematological malignancies has motivated similar studies using TCR-like mAbs [42]. The HLA-A2/WT1 mAb ESK1 has been evaluated both as a BiTE and as CAR T cell [129,130]. BiTEs normally target cell-surface proteins that are expressed on cell subsets and at high density, such as the CD19 BiTE (Blinatumomab/Blincyto) that is approved by the U.S. Food and Drug Administration (FDA) for treatment of ALL [131]. However, despite the low antigen density of specific pMHCs, an ESK1-BiTE was still effective in clearing tumor cells in NSG mouse models of three cancers [129]. Interestingly, an in vitro culture assay used to assess specific autologous tumor recognition revealed that the ESK1-BiTE induced a polyclonal activation of patient T cells against non-WT1 tumor epitopes. Such a scenario in vivo could possibly provide a broader and more effective anti-tumor immune response by epitope spreading. Similarly to the ESK1-BiTE, the ESK1 CAR (denoted WT1-28z CAR) was also able to kill tumor cells and enhance survival of mice, in particular with co-expression of IL-12 [130]. This proof-of-concept extended previous in vitro CAR studies targeting WT1-presenting tumor cells [67].

Engineering of TCR-like mAbs to increase valency or affinity has been shown to enhance the therapeutic potential. One such example is the soluble protein format “dimeric bispecific T cell-engaging tandem scFv antibody” (DiBsAb), that enables bivalent engagement of both the target cell and the effector cell. Targeting of tumor cell lines presenting the EBV-derived epitope LMP2A on HLA-A2 demonstrated more potent in vitro activity of the affinity-matured 38-2 DiBsAb compared to the parent clone 38 [70]. Additionally, the high-affinity variant slightly prolonged the survival of double knock-out (DKO) (Rag2^-/-^ Il2rg ^-/-^) mice injected with EBV^+^ tumor cells in a xenograft model compared to the 38 DiBsAb. However, the increase in affinity resulted in cross-reactivity towards HLA-A2-positive cells and cells loaded with homologous peptides. Thus, the mother clone was concluded to have a superior safety profile. Analogous to the previous example, the affinity-matured HLA-A2/WT1 TCR-like mAb Q2L (scFv-hIgG1 Fc fusion) also exhibited improved cytotoxic capacity in in vitro ADCC assays as well as significantly reducing tumor burden in xenografted DKO mice compared to the mother clone [68]. Also, here, there were some evidence that the increased affinity resulted in some cross-reactivity.

### 6.2. Autoimmunity

The use of TCR-like mAbs in therapeutic intervention of autoimmunity offers a unique treatment avenue by preventing pathogenic T-cell activation (Figure 2). This patient group usually relies on life-long treatment after disease onset, and current treatments are not disease-specific and frequently result in low and heterogeneous efficacy as well as unpredictable and burdening side effects. Despite these shortcomings, broadly acting mAb therapy aiming at modulation of inflammatory signaling pathways, or blocking and depletion of entire immune cell populations, are used with acceptable clinical effect [132]. Thus, the specific targeting of pathogenic autoreactive cells, while sparing protective immune cells and non-diseased tissues, would be a huge clinical improvement.

As early as in 1991, Aharoni et al. reported that hybridoma-derived murine IgM mAbs against I-A^s^ presenting an MBP peptide could provide antigen-specific therapeutic benefit against EAE in mice [84]. Indeed, later studies targeting the B:9–23 insulin epitope presented on I-A^g7^ showed that the TCR-like mIgG1 mAb287 delayed disease onset in diabetic NOD mice as an experimental model for type 1 diabetes (T1D) when administered at both early or late stages of disease [81]. Surprisingly and importantly, targeting of this single MHCII epitope resulted in a pleiotropic disease-specific effect, where not only islet cell infiltration of insulin-specific CD4 T was prevented, but also B cells, as well as CD4 and CD8 T cells of other specificities. Importantly, there was no indication of global immune suppression. The precise mode of action of mAb287 remains unclear, but the authors speculate that selective deletion of target APCs, which in addition to presenting the B:9–23 insulin epitope also present other epitopes, could lead to an overall depletion of pMHCs [81,133]. Another possible explanation for the observed effect could be induction of specific CD4 suppressor cells in line with earlier observations using pan-MHCII mAbs [134]. That targeting of a single epitope for disease-specific modulation of autoimmunity without perturbing the function of non-disease-associated T-cell specificities is possible, is further supported by similar results obtained by Dahan and colleagues in the humanized HLA-DR4.1/GAD65 model of T1D using the TCR-like mAb G3H8 [89].

Recently, the potential of CAR T cells as a treatment strategy for autoimmunity was explored in diabetic NOD mice. Here, CAR T cells were constructed based on mAb287 and were shown to delay onset of disease, as had been observed by the parent mAb [81,133]. Notably, whereas the parent mAb relied on weekly injections, the CAR T cells were administered once and still showed efficacy [81]. However, even though the effect was not durable, the study demonstrated proof-of-principle in targeting peptide-presenting cells as an intervention of autoimmunity.

## 7. Summary and Future Directions

Method development affecting TCR-like mAb generation now makes these precision molecules readily available at a realistic scale both as research tools and for therapeutic evaluation. They have been proven to be powerful reagents to understand T-cell responses in both animal models and human disease, and extend the scope of conventional immunotherapy by allowing for truly disease-specific intervention in cancer, infection and autoimmunity by targeting of defined epitopes. This contrasts with conventional mAbs used in immunotherapy that target lineage markers and thus entire cell populations. The immediate inherent limitation of TCR-like mAbs is the dependence on specific HLA variants, which potentially narrows the patient group suitable for therapy. Most TCR-like mAbs are therefore focused on peptides presented on frequently expressed HLA molecules. The broad use and versatility of the antibody format in conjunction with the current improvements in personalized therapeutic approaches, render these limitations in HLA restriction a minor obstacle. Further, several autoimmune diseases, such as narcolepsy, CeD, MS and RA, are strongly associated with specific HLA variants, making TCR-like mAbs targeting such complexes broadly applicable [31].

Comparisons of TCR-like mAbs and TCRs show that the pMHC binding mode is not necessarily the same, which has consequences for how they may differentially sample the MHC ligandome. As antibodies do not pass through thymic selection dampening putative cross-reactivity, this aspect needs to be better characterized and managed for their safe use as therapeutics. At present, none of the reported TCR-like mAb specificities are in clinical evaluation, and have so far only been studied in vitro or in mouse models with limited or no HLA diversity [89,124,128,129]. Here, the relevant MHC is expressed on the transferred cells only, possibly masking any true toxicity effects resulting from peptide cross-reactivity. However, given the advances and lessons learnt in the engineered TCR field, our understanding on how to manage these aspects has reached a level that should be immediately transferable also to TCR-like mAb specificities [17,135].

The main prevailing hurdle to effectively integrate TCR-like mAbs in the growing arsenal of precision therapy is the availability of clinically validated and relevant targets [136,137]. Current efforts in consolidation of big data into a readily accessible format, such as the SystemMHC atlas for the MHC ligandome, together with improved MHC ligandome algorithms and discovery tools, should greatly facilitate future progress in this area [29,138,139,140]. Here, the autoimmune field stands out as the most challenging due to the inherent complexity of these diseases [136]. However, recent insights in preclinical models of RA, T1D and MS suggest that single epitope targeting can be used for highly disease-specific interventions [141,142]. The observation that this may also be achieved using the well-established antibody format where suppression of the response to only one major autoimmune epitope was sufficient to change the course of the disease should indeed encourage further exploration of this intervention path using TCR-like mAbs [81,89].

## Figures and Tables

**Table 1:** T-cell receptor (TCR)-like monoclonal antibodies (mAbs) against peptide–major histocompatibility class I (pMHCI).

Antigen	Epitope	MHC	Clone	Indication	Affinity ^1^	Selection Method	References
PR8	NA	H-2D^k^/H-2K^b^	NA	Infection	ND	Hybridoma	[35]
SV40	NA	H-2K^b^		Infection	ND	Hybridoma	[36]
NP	NA	K^d^	X5.3.7	Infection	ND	Hybridoma	[37]
HA	FESTGNLI	K^k^	Fab13.4.1	Infection	50 nM	Immunization/phage	[38]
pOV8	SIINFEKL	K^k^	25-D1.16 ^2^	Model antigen	ND	Hybridoma	[39]
MAGE-A1	EADPTGHSY	HLA-A*0101	G8	Cancer	250 nM	Phage	[40]
MAGE-A1	EADPTGHSY	HLA-A*0101	Hyb3 ^2,^^3^	Cancer	14 nM	Phage	[41,42]
MIF	FLSELTQQL	HLA-A*0201	RL21A	Cancer	24.4 nM	Hybridoma	[43]
MUC1	LLLTVLTVV	HLA-A*0201	M3A1, M3B8	Cancer	ND	Phage	[44]
gp100	KTWGQYWQV	HLA-A*0201	G2D12,	Cancer	ND	Phage	[45]
gp100	ITDQVPFSV	HLA-A*0201	1A7	Cancer	ND	Phage	[45]
gp100	YLEPGPVTA	HLA-A*0201	2F1	Cancer	ND	Phage	[45]
gp100	ITDQVPFSV	HLA-A*0201	GPA7 ^4^	Cancer	180 nM	Phage	[46]
gp100	IMDQVPFSV	HLA-A*0201	G1	Cancer	ND	Phage	[47]
hTERT	ILAKFLHWL	HLA-A*0201	4A9, 4G9	Cancer	ND	Phage	[48]
hTERT	RLVDDFLLV	HLA-A*0201	3G3, 3H2	Cancer	ND	Phage	[48]
HTLV-1	LLFGYPVYV	HLA-A*0201	T3E3, T3F2	Infection	ND	Phage	[49]
M1	GILGFVFTL	HLA-A*0201	M1-A2, M1-D1, M1-D12, M1-G8	Infection	ND	Phage	[50]
NY-ESO-1	SLLMWITQC	HLA-A*0201	3M4E5, 3M4F4 ^2^	Cancer	46–95 nM	Phage	[51,52]
NY-ESO-1	SLLMWITQC	HLA-A*0201	T1 ^3^	Cancer	2–4 nM	Phage	[52]
MelanA/MART-1	EAAGIGILTV	HLA-A*0201	E5, H4	Cancer	ND	Phage	[53]
MelanA/MART-1	ELAGIGILTV	HLA-A*0201	2M3F11, 3N4E9, 2N4B4, 3N4B5	Cancer	ND	Phage	[53]
MelanA/MART-1	EAAGIGILTV	HLA-A*0201	CAG10, CLA12	Cancer	ND	Phage	[54]
hCGβ	GVLPALPQV	HLA-A*0201	RL4B/3.2G1	Cancer	ND	Hybridoma	[55]
hCGβ	GVLPALPQV	HLA-A*0201	1B10	Cancer	ND	Hybridoma	[56]
hCGβ	TMTRVLQGV	HLA-A*0201	3F9	Cancer	ND	Hybridoma	[56]
MAGE3	FLWGPRALV	HLA-A*0201	7D4	Cancer	ND	Hybridoma	[57]
PR1	VLQELNVTV	HLA-A*0201	8F4	Cancer	9.9 nM	Hybridoma	[58]
P68 RNA Helicase	YLLPAIVHI	HLA-A*0201	RL6A	Cancer	0.42 nM	Hybridoma	[59]
HER2/Neu	KIFGSLAFL	HLA-A*0201	1B8	Cancer	ND	Hybridoma	[60]
HER2/Neu	KIFGSLAFL	HLA-A*0201	fE75	Cancer	59 nM	Phage	[61]
HER2/Neu	KIFGSLAFL	HLA-A*0201	RL1B	Cancer	2.69 nM	Hybridoma	[62]
Calreticulin	MLSVPLLL	HLA-A*0201	fML	Cancer	79 nM	Phage	[61]
PRAME	ALYVDSLFFL	HLA-A*0201	Pr20	Cancer	ND	Phage	[63]
AFP	FMNKFIYEI	HLA-A*0201	ET1402L1	Cancer	ND	Phage	[64]
WT1	RMFPNAPYL	HLA-A*0201	ESK1 ^2^	Cancer	ND	Phage	[65,66]
WT1	RMFPNAPYL	HLA-A*0201	F2, F3	Cancer	400, 30 nM	Phage	[67]
WT1	RMFPNAPYL	HLA-A*0201	Clone45	Cancer	263 nM	Phage	[68]
WT1	RMFPNAPYL	HLA-A*0201	Q2L ^3,5^	Cancer	3 nM	Yeast	[68]
LMP1	YLLEMLWRL	HLA-A*0201	L1	EBV-cancer	1.85 nM	Hybridoma	[69]
LMP2A	CLGGLLTMV	HLA-A*0201	L2	EBV-cancer	6.98 nM	Hybridoma	[69]
EBNA1	FMVFLQTHI	HLA-A*0201	E1	EBV-cancer	6.02 nM	Hybridoma	[69]
LMP2A	CLGGLLTMV	HLA-A*0201	38	EBV-cancer	ND	Phage	[70]
LMP2A	CLGGLLTMV	HLA-A*0201	38-2 ^3^	EBV-cancer	ND	Phage	[70]
KRAS	KLVVVGAVGV	HLA-A*0201	D10	Cancer	ND	Phage	[71]
KRAS	KLVVVGAVGV	HLA-A*0201	D10-7 ^3^	Cancer	ND	Phage	[71]
EGFR	KITDFGRAK	HLA-A3	C9	Cancer	ND	Phage	[71]
TARP	FLRNFSLML	HLA-A*0201	D2	Cancer	ND	Phage	[72]
HSP16	GILTVSVAV	HLA-A*0201	A2/Ab(clone3) ^4^	Infection	ND	Phage	[73]
eIF4G	VLMTEDIKL	HLA-A*0201	4F7	Infection	ND	Hybridoma	[74]
HA-1H	VLHDDLLEA	HLA-A*0201	#131	Cancer	19.9 nM	Phage	[75]
Tyrosinase	YMDGTMSQV	HLA-A*0201	TA2	Cancer	ND	Phage	[76]
p53	RMPEAAPPV	HLA-A*0201	T1-116C	Cancer	ND	Hybridoma	
p53	RMPEAAPPV	HLA-A*0201	T1-29D and T1-84C	Cancer	ND	Hybridoma	[77]
p53	GLAPPQHLIRV	HLA-A*0201	T2-108A, T2-2A, T2-116A	Cancer	ND	Hybridoma	[77]

In case of multiple candidate mAbs, the lead candidates are described in the Table. NA; not available. ND; not determined. ^1^ Affinity values determined by 1:1 binding using surface plasmon resonance. ^2^ Available co-crystal structure with pMHC. ^3^ Affinity matured variant. ^4^ Single domain antibody (Dab), based on llama V_H_H or human VH3-23/DP47. ^5^ Docking model of Fv onto pMHC.

**Table 2:** T-cell receptor (TCR)-like monoclonal antibodies (mAbs) against peptide–major histocompatibility class II (pMHCII).

Antigen	Epitope	MHC	Clone	Indication	Affinity ^1^	Selection method	References
2W	EAWGALANWAVDSA	I-A^b^	W6	Infection	3.4 nM	Hybridoma	[78]
Eα	ASFEAQGALANIAVDKA	I-A^b^	Y-Ae	Self-peptide	0.48 nM	Hybridoma	[79]
LACK	ICFSPSLEHPIVVSGSWD	I-A^d^	2C44	Infection	ND	Hybridoma	[80]
insulin	HLVERLYLVCGEEG	I-A^g7^	mAb287	Autoimmunity	130 nM	Hybridoma	[81]
p63	RTRPLWVRME	I-A^g7^	FS1	Autoimmunity	0.02 nM	Hybridoma	[78]
HEL	NTDGSTDYGILQINSR	I-A^k^	B6Ge1	Model antigen	ND	Hybridoma	[82]
HEL	KGTDVQAWIRGCRL	I-A^k^	D8H21	Model antigen	ND	Hybridoma	[82]
HEL	DGSTDYGILQINSRW	I-A^k^	Aw3.18	Model antigen	12.4 nM	Hybridoma	[83]
MBP	VHFFKNIVTPRTP	I-A^s^	B-7-1, B-18-7, C34-72	Autoimmunity	ND	Hybridoma	[84]
MCC	IAYLKQATK	I-E^k^	D4,G32,G35	Model antigen	700 nM	Hybridoma	[85]
HLA-A2	SDWRFLRGYHQYA	HLA-DR1	UL-5A1	Self-peptide	ND	Hybridoma	[86]
MBP	ENPVVHFFKNIVTPR	HLA-DR2b	MK16	Autoimmunity	ND	Immunization/phage	[87]
MOG	MEVGWYRPPFSRVVHLYRNGK	HLA-DR2b	2E4, 1F11, 3A3, 3H5, 2C3	Autoimmunity	30–150 nM	Phage	[88]
GAD65	NFFRMVISNPAAT	HLA-DR4.1	G1H12, G3H8, D2	Autoimmunity	64 nM, 104 nM	Phage	[89]
HC gp-39	RSFTLASSETGVG	HLA-DR4.1	12A	Autoimmunity	ND	Hybridoma	[90]
Gluten	QLQPFPQPELPY	HLA-DQ2.5	106, 107 ^2^	Autoimmunity	70 nM, 100 nM	Phage	[91]

In case of multiple candidate mAbs, the lead candidates are described in the Table. NA; not available. ND; not determined. ^1^ Affinity values determined by 1:1 binding using surface plasmon resonance or bio-layer interferometry. ^2^ Docking model of Fv onto pMHC.
